# A Triple Pedicle, Near-Total Thigh Flap Supercharged With the Flow-Through Technique

**Published:** 2016-01-13

**Authors:** Mofiyinfolu Sokoya, Frederic W.-B. Deleyiannis

**Affiliations:** Department of Otolaryngology/Head and Neck Surgery, University of Colorado School of Medicine, Aurora

**Keywords:** ALT, total thigh free flap, supercharging, flow-through technique, tensor fascia latae flap

## Abstract

This is the first instance where the technique of supercharging a flap with a second pedicle, combined with a third pedicle using the flow-through technique, is described in literature. We present the case of a 66-year-old man with multiple recurrent squamous and basal cell carcinomas of the face. He underwent a wide local excision resulting in a 20 × 26-cm defect. This was reconstructed with near-total thigh free flap. Three separate pedicles providing independent perforators to the medial, posterior-lateral, and anterolateral thigh from the superficial femoral artery, directly from the profunda femoris artery, and from the descending branch of the lateral circumflex artery were harvested. We expand upon the technique of supercharging a flap by not only anastomosing 3 separate pedicles but also using the flow-through technique to provide inflow and outflow to the third pedicle.

The overall size of an anterolateral thigh (ALT) free flap is dependent on the number and location of skin perforators. Given that the perforators of an ALT flap routinely arise sequentially from the descending branch of the lateral circumflex artery as it descends down the thigh, it is likely that a long ALT flap can be more reliably harvested than a wide ALT flap.^1^ Deleyiannis et al^2^ recently described the concept of supercharging and venous augmenting the flap as an option for increasing the width of the flap based on separate vascular pedicles from the superior, medial, and/or posterior-lateral thigh. In the present case, we expand this option by not only anastomosing 3 separate pedicles but also using the flow-through technique to provide inflow and outflow to the third pedicle.

## CASE

A 66 year old man with a history of multiple recurrent squamous and basal cell carcinomas of the head and neck presented for palliative therapy. He had a history of radiation therapy for acne as a child, as well as chronic sun exposure, and was chronically immunosuppressed secondary to the use of mycophenolic acid (CellCept) for myasthenia gravis. He had previously undergone multiple Mohs surgical procedures, adjuvant chemoradiotherapy, and multiple reconstructions with skin grafts and local flaps. His preoperative examination showed a large painful right cheek skin ulceration, with numerous smaller ulcerations interspersed by previous reconstructions of split-thickness skin grafts, involving the postauricular skin, temple, and upper neck. He had complete paralysis of his right face. With the goals of pain relief and improved local hygiene, he underwent a radical excision of the entire right face, right ear, and upper neck.

**Figure 1:**
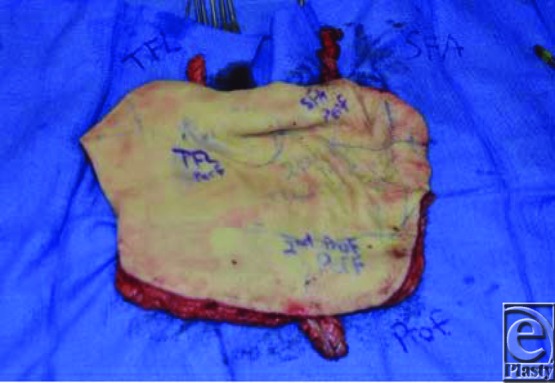
pedicles providing independent perforators to the medial, posterior-lateral, and anterolateral thigh from the superficial femoral artery, directly from the profundus femoris artery, and from the descending branch of the lateral circumflex artery.

**Figure 2:**
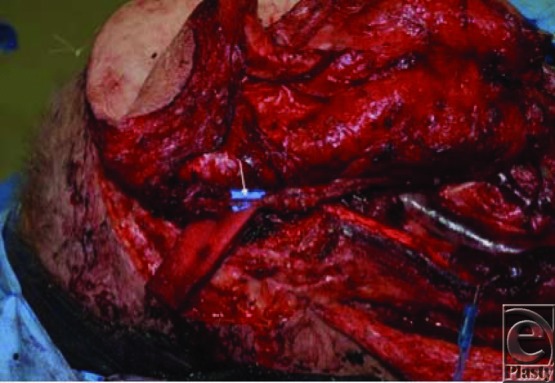
Three arterial and 5 venous anastomoses were done (see text). The arrow indicates the flow-through anastomosis (ie, flow going through the facial artery to the descending lateral circumflex artery and then to the perforator originating from the profunda femoris artery.

**Figure 3:**
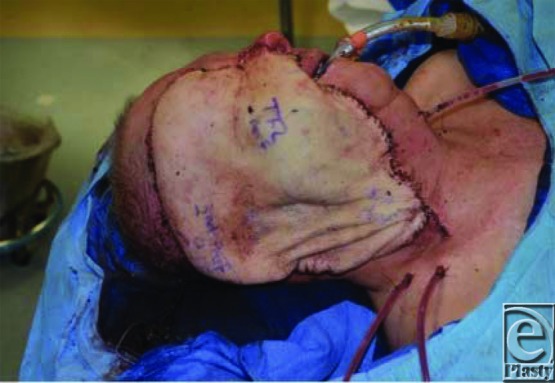
Appearance after anastomosis and inset.

To reconstruct the cutaneous defect, which measured 20 × 26 cm, a near-total thigh free flap was designed. Three separate pedicles providing independent perforators to the medial, posterior-lateral, and anterolateral thigh from the superficial femoral (SF) artery, directly from the profunda femoris (PF) artery, and from a transverse branch coming off the descending branch of the lateral circumflex and traversing through the tensor fasciae latae muscle (Fig 1). Three arteries and 5 venous anastomoses were then performed as follows: descending branch of the lateral circumflex (DLC) artery/vein to the facial artery and vein ×2; SF artery/vein to the superior thyroid artery and external jugular vein; PF artery/vein to the distal end of the DLC artery and vein, that is, flow-through anastomoses: inflow going from the facial artery to the DLC and then to the PF branch (Fig 2). The flap healed without venous congestion or arterial insufficiency (Fig 3).

## DISCUSSION

The 3 pedicles prepared for this reconstruction could have each independently supported different thigh free flaps with distinct zones of perfusion. By combining the 3 pedicles into 1 large thigh flap, the dimensions of the flap could reliably be increased to reconstruct this massive defect with the lowest risk of ischemia or venous congestion. The use of the flow-through technique enabled the selection of 1 less cervical artery and vein for inflow and outflow. The selection of which pedicle to perfuse with the flow-through technique will depend on the orientation of the pedicles to each other once the flap is inset, as well as the lengths of each pedicle. If a flow-through flap is planned, the DLC pedicle should be dissected well past the takeoff point of the last skin perforator so that adequate length can be achieved to place this pedicle next to either the SF or PF pedicle.

The main indication for a triple pedicle thigh flap is an extensively wide or long defect that cannot be reliably closed with a single perforator ALT flap or an ALT flap supercharged only with 1 additional pedicle. What remains uncertain is the maximum dimensions of an ALT flap that can be harvested in both these situations.^3^

## SUMMARY

Supercharging a thigh flap with a second pedicle and with third pedicle perfused by the flow-through technique may be useful when confronted with a massive cutaneous defect and with a limited choice of recipient cervical vessels.
